# Interferon-Induced HERC5 Inhibits Ebola Virus Particle Production and Is Antagonized by Ebola Glycoprotein

**DOI:** 10.3390/cells10092399

**Published:** 2021-09-13

**Authors:** Ermela Paparisto, Nina R. Hunt, Daniel S. Labach, Macon D. Coleman, Eric J. Di Gravio, Mackenzie J. Dodge, Nicole J. Friesen, Marceline Côté, Andreas Müller, Thomas Hoenen, Stephen D. Barr

**Affiliations:** 1 Department of Microbiology and Immunology, Schulich School of Medicine and Dentistry, Western University, Dental Sciences Building Room 3007, London, ON N6A 5C1, Canada; epaparis@uwo.ca (E.P.); nhunt8@uwo.ca (N.R.H.); dlabach@uwo.ca (D.S.L.); mcolem5@uwo.ca (M.D.C.); edigravi@uwo.ca (E.J.D.G.); mdodge@uwo.ca (M.J.D.); nfriese5@uwo.ca (N.J.F.); 2 Department of Biochemistry, Microbiology, and Immunology, Ottawa Institute of Systems Biology, University of Ottawa, Roger-Guindon Hall Room 4214, Ottawa, ON K1H 8M5 , Canada; Marceline.Cote@uottawa.ca; 3 Friedrich-Loeffler-Institut, Institute of Molecular Virology and Cell Biology, Südufer 10, 17493 Greifswald—Insel Riems, Germany; andreas.mueller@seracell.de (A.M.); Thomas.Hoenen@fli.de (T.H.)

**Keywords:** Ebola virus, Marburg virus, HERC5, antiviral, interferon

## Abstract

Survival following Ebola virus (EBOV) infection correlates with the ability to mount an early and robust interferon (IFN) response. The host IFN-induced proteins that contribute to controlling EBOV replication are not fully known. Among the top genes with the strongest early increases in expression after infection in vivo is IFN-induced HERC5. Using a transcription- and replication-competent VLP system, we showed that HERC5 inhibits EBOV virus-like particle (VLP) replication by depleting EBOV mRNAs. The HERC5 RCC1-like domain was necessary and sufficient for this inhibition and did not require zinc finger antiviral protein (ZAP). Moreover, we showed that EBOV (Zaire) glycoprotein (GP) but not Marburg virus GP antagonized HERC5 early during infection. Our data identify a novel ‘protagonist–antagonistic’ relationship between HERC5 and GP in the early stages of EBOV infection that could be exploited for the development of novel antiviral therapeutics.

## 1. Introduction

Ebola virus (EBOV) is a member of the *Filoviridae* family of single-stranded negative-sense RNA viruses with a filamentous morphology. EBOV infection results in severe hemorrhagic fever and can lead to death 6-16 days after the onset of symptoms in up to 90% of cases, making EBOV one of the most virulent pathogens to infect humans [1]. Studies involving primate models, and human studies carried out during the 2013–2016 outbreak, showed that EBOV exposure results in an early and robust immune response, largely characterized by the up-regulation of IFN-stimulated genes [2,3,4,5,6,7,8,9,10,11,12]. A contributing factor to the pathophysiology of EBOV infection is the ability of the virus to evade the host IFN response [7,13,14,15,16]. Using in vitro models of infection, it was shown that EBOV is able to evade the innate immune response through various IFN antagonisms, notably involving VP24 and VP35 proteins [17,18,19]. The key mediators of this early cellular IFN response to EBOV and how EBOV withstands this early response are not fully characterized.

Restriction factors are key intrinsic mediators of the early IFN response and potently inhibit different steps in the life cycle of evolutionarily diverse viruses in the absence of viral antagonists [20]. Bone marrow stromal cell antigen 2 (BST-2)/tetherin is one such factor that potently inhibits the release of EBOV from cells by tethering virions to the surface of cells [21,22]. This inhibition is counteracted by EBOV GP [23,24,25,26]. IFN-inducible trans-membrane proteins 1–3 (IFITM1–3) comprise another family of factors that restrict the cellular entry of EBOV, although an EBOV antagonist to these proteins has yet to be identified [27,28]. HECT and RCC1-like containing domain 5 (HERC5) are some of the genes with the strongest early increases in expression in multiple tissues after EBOV infection [3,5,6,29]. HERC5 is an evolutionarily ancient restriction factor that inhibits the replication of diverse viruses [30,31,32,33,34,35,36]. By virtue of its C-terminal HECT domain, HERC5 is the main cellular E3 ligase for conjugating ISG15 to substrates and localizes to polyribosomes to modify newly translated viral proteins, thereby disrupting key aspects of viral particle production [31,35,37,38]. E3 ligase-independent antiviral activity has also been demonstrated towards HIV-1, where it inhibits the nuclear export of incompletely-spliced viral RNAs by a mechanism requiring its N-terminal RCC1-like domain (RLD) [30].

Here, we examined the antiviral activity of HERC5 towards EBOV VLP production and replication. We identified a novel E3 ligase-independent mechanism by which HERC5 inhibits viral particle production involving the depletion of EBOV mRNAs. In addition, we demonstrated that EBOV GP antagonizes HERC5 activity and rescues EBOV VLP production and replication.

## 2. Materials and Methods

### 2.1. Cell Lines

293T and HeLa cells were obtained from American Type Culture Collection. 293T ZC3HAV1 (ZAP) knockout cells were obtained from Dr. Takaoka (Hokkaido University, Japan) via Dr. Li (University of California, Los Angeles, CA, USA) and Dr. MacDonald (The Rockefeller University, New York, NY, USA). Cells were maintained in standard growth medium (Dulbecco’s Modified Eagle’s Medium (DMEM)), supplemented with 10% heat-inactivated Fetal Bovine Serum (FBS), 100 U/mL Penicillin and 100 μg/mL Streptomycin) at 37 °C with 5% CO_2_.

### 2.2. Plasmids, Transfections, Antibodies and Quantitative Western Blotting

Expression plasmids carrying FLAG-tagged HERC5, HERC5-ΔRLD, HERC5-ΔHECT and HERC5-C994A, and HERC4 have been described previously [36]. The plasmid carrying FLAG-tagged RLD only (pFLAG-RLDonly) was generated by standard restriction enzyme cloning of the HERC5 RLD (containing a 3′ stop codon) into p3xFLAG-CMV-10 (Sigma). The promoterless empty vector plasmid pGL3, pEGFP-C1 (pEGFP) and pZAP (short isoform) were obtained from Promega, Clontech and Dharmacon, respectively. pLKO.1/scrambled shRNA and pLKO.1/HERC5 shRNA were previously described [30,31]. VP40 and GP were cloned into p3xFLAG-CMV-10 (Sigma) to generate pFLAG-VP40, pFLAG GP and pEGFP-C1 (containing a CMV promoter) (Clontech) to generate pVP40-EGFP using standard restriction enzyme cloning. EBOV expression plasmids: pCAGGS plasmids (containing a CMV enhancer, chicken beta-actin promoter and beta-actin intron sequence) carrying only EBOV (Zaire) VP40, VP30, VP35, L, NP, or GP were obtained from Dr. Kawaoka (University of Wisconsin) [39]. Plasmids for the trVLP assay were provided by Dr. Hoenen (Friedrich-Loeffler-Institut, Germany): Plasmids carrying NP, VP35, VP30, L, Tim-1, T7 and the tetracistronic minigenomes (p4cisvRNA-hrLuc, p4cis-vRNA-EGFP) have been previously described [40,41]. All EBOV gene sequences in the minigenomes and plasmids carrying NP, VP35, VP30, and L originated from the Zaire EBOV isolate *H. sapiens*- tc/COD/1976/Yambuku-Mayinga. The EBOV GP and MARV GP expression plasmids were kind gifts of Dr. Cunningham (Brigham and Women’s Hospital) [42,43]. Transfections were performed using Lipofectamine 2000 (Invitrogen) per manufacturer’s instructions unless otherwise stated. Co-transfections of HERC5 plasmids with pVP40 were performed at a ratio of 10:1, respectively, unless otherwise noted. VP40 VLPs were purified from cell supernatants by centrifugation over a 20% sucrose cushion at 21,000× *g* for 2 h. Cell lysates and VP40 VLP pellets were subjected to quantitative Western blot analyses using LI-COR, as previously described [30]. Densitometric analysis was performed using ImageJ 1.53e 64-bit version software. Antibodies: Anti-FLAG was purchased from Sigma, anti-ZAP from AbCam (Cat. #ab154680), anti-VP40 from GeneTex (Cat. #GTX134034), anti-MARV GP from Alpha Diagnostic International (Cat. #MVGP12-A), anti-EBOV GP from Bio-Techne (Cat. #MAB9016), anti-β-actin from Rockland, anti-EGFP from Clontech and anti-GAPDH (clone 6C5) from EMD/Millipore. 

### 2.3. Confocal Immunofluorescence Microscopy

HeLa cells were cultured in 12-well plates on 18 mm coverslips and co-transfected with either pFLAG-HERC5 and pVP40-EGFP (10:1 ratio) or pGL3 and pVP40-EGFP (10:1 ratio). Twenty-four hours after transfection, the coverslips containing the cells were washed twice with PF buffer (1× PBS + 1% FBS), fixed for 10 min in 1× PBS containing 4% formaldehyde and 2% sucrose, permeabilized in 1× PBS containing 0.1% Triton X 100 (Sigma) and then washed twice more with PF buffer. Coverslips were incubated with primary antibody rabbit anti-FLAG (1:500 dilution) for 1 h, washed 3× with PF buffer and incubated with either secondary antibody anti-rabbit 594 (1:1000) for 1 h. Coverslips were washed 3×, incubated in Hoechst 33342 (1:10,000 dilution) (Life Technologies) for 5 min and washed 6× with PF buffer. Coverslips were then mounted on glass slides with 10 µL Vectashield mounting media (Vector Laboratories Inc., Burlingame, CA, USA) and sealed with nail polish. Confocal micrographs were obtained using a Leica TCS SP8 (Leica Microsystems) microscope, and Leica Application Software X was used for image acquisition.

### 2.4. Transmission Electron Microscopy

Cells were co-transfected with empty vector or pFLAG-HERC5 and pVP40-EGFP at a 10:1 ratio. After 48 h, cells were resuspended in media, fixed in 2.5% glutaraldehyde in 0.1 M sodium cacodylate (pH 7.4) for 2 h, and washed 3× in 0.1 M sodium cacodylate. Cells were pelleted and fixed with 2% osmium tetroxide in sodium cacodylate. After ~1 h in the dark, cells were washed 3× in ddH_2_O. Water was discarded, and samples were left at 4 °C overnight. Samples were dehydrated by adding 1 mL 20% acetone in ddH_2_O, mixed and incubated for 10 min at room temperature. Cells were pelleted, acetone removed, and the procedure was repeated with 50%, 70%, 90%, 100%, 100% and 100% acetone. Cells were embedded in resin by adding 1 mL of a 2:1 mix of acetone:resin (Epon) and incubated for ~4 h at room temperature in a rotating tube shaker. Cells were pelleted, acetone:resin mix was discarded and repeated with a 1:1 mix overnight, 1:2 mix overnight, and finally, resin only overnight. Samples were cut in 70 nm slices using a Sorval Ultracut ultramicrotome and placed onto 400 mesh nickel grids (Embra). Grids were placed on drops of 2% uranyl acetate in ddH_2_O to stain for 20 min in the dark and washed 5–6× in ddH_2_O for 1 min. Samples were then stained in drops of Sato’s lead citrate (5 mM calcined lead citrate, 11 mM lead nitrate, 11 mM lead acetate, 95 mM sodium citrate) for 1 min and washed using ddH_2_O. Samples were imaged using a Phillips CM10 Transmission Electron Microscope. The AMT Advantage digital imaging system was used for image acquisition. 

### 2.5. Quantitative PCR

The total RNA was extracted using the PureLink RNA mini kit (Ambion, Life Technologies). Using the M-MLV reverse transcriptase and Oligo(dT) primers (Eurofins), 500 ng of RNA was reverse transcribed to cDNA. Prior to qPCR, cDNA samples were diluted 1:5 with water. Each PCR reaction consisted of 10 μL of SYBR Green Master Mix, 1.6 μL of gene-specific primers (0.8 μL of 10 μM forward primer and 0.8 μL of 10 μM reverse primer), 4 μL of diluted cDNA, and water to a total volume of 20 μL. Quantification of endogenous mRNA was run on the QuantStudio5 qPCR machine (Applied Biosystems) under the following cycling conditions: 2 min at 95 °C and 40 cycles of 5 sec at 95 °C, 10 s at 60 °C, and 20 s at 72 °C. The QuantStudio Design and Analysis Desktop Software (version 1.4) was used to determine the C_T_ for each PCR reaction. Primer pairs were as follows: HERC5- (fwd: 5′ ATG AGC TAA GAC CCT GTT TGG 3′; rev: 5′ CCC AAA TCA GAA ACA TAG GCA AG 3′); ZAP- (fwd: 5′ CGCTTAATGGTAGCTGCAGC 3′; rev: 5′ CCTACAGAACAGAGGTGGATTCC 3′); GAPDH- (fwd: 5′ CAT GTT CGT CAT GGG TGT GAA CCA 3′; rev: 5′ AGT GAT GGC ATG GAC TGT GGT CAT 3′); EGFP- (fwd: 5′ GACAACCACTACCTGAGCAC 3′; rev: 5′ CAGGACCATGTGATCGCG3′); EBOV VP40- (fwd: 5′GCTTCCTCTAGGTGTCGCTG3′ ; rev 5′GGTTGCCTTGCCGAAATGG3′); EBOV GP- (fwd: 5′GTGAATGGGCTGAAAACTGC3′ ; rev 5′CCGTTCCTGATACTTTGTGC3′); EBOV VP30- (fwd: 5′CCAGACAGCATTCAAGGG3′; rev 5′GCTGGAGGAACTGTTAATGG3′); EBOV VP35- (fwd: 5′CGACTCAAAACGACAGAATGC3′ ; rev 5′GGTTTGGCTTCGTTTGTTGC3′); EBOV NP- (fwd: 5′GCCAACTTATCATACAGGCC3′ ; rev 5′CCAAATACTTGACTGCGCC3′); EBOV L- (fwd: 5′CCTAGTCACTAGAGCTTGCG3′ ; rev 5′GGCTCAACAGGACAGAATCC3′). To ensure no carry-over of DNA into each total purified RNA sample, 100 ng of RNA was used directly as a template without reverse transcription for qPCR using the primer sets described above.

### 2.6. trVLP Assay

Expression plasmids carrying tim-1, T7, NP, VP35, VP30, L, and the tetracistronic minigenome (p4cis-vRNA-hrLuc) carrying luciferase, VP40, GP and VP24 have been previously described [40,44]. trVLP assays were performed as previously described, with the following changes [40,41]. Passage zero (p0) cells were seeded in 12-well plates and transfected at 50% confluency using Transit LT-1 (Mirus Bio LLC, Madison, WI, USA) with expression plasmids carrying T7-polymerase (125 ng; all amounts per well), the viral proteins NP (62.5 ng), VP35 (62.5 ng), VP30 (37.5 ng), L (500 ng), a tetracistronic minigenome (125 ng), and Firefly luciferase (100 ng) following the manufacturer’s instructions. Twenty-four hours prior to infection of p1, p2, p3 and p4 cells, target cells were pre-transfected with expression plasmids carrying NP (62.5 ng), VP35 (62.5 ng), VP30 (37.5 ng), L (500 ng), Tim-1 (125 ng) and either HERC5 (125 ng) or empty vector (125 ng). 

### 2.7. Cell Viability Assay

293T cells were co-transfected with pFLAG-VP40, GFP-VP40 or GFP alone, as well as increasing concentrations of pFLAG-HERC5 or empty vector control plasmid. Forty-eight hours post-transfection Cell Counting Kit-8 (CCK-8) (GLPBIO) was used to measure cell viability as per the manufacturer’s instructions.

### 2.8. Statistical Analyses

GraphPad Prism v9 was used for all statistical analyses stated in the text. *p* values and statistical tests used are stated in the text where appropriate. *p* values less than 0.05 were deemed significant. Quantification of immunogold labelling for statistical analysis was performed as described [31].

## 3. Results

### 3.1. HERC5 Inhibits EBOV trVLP Replication

**Figure 1:**
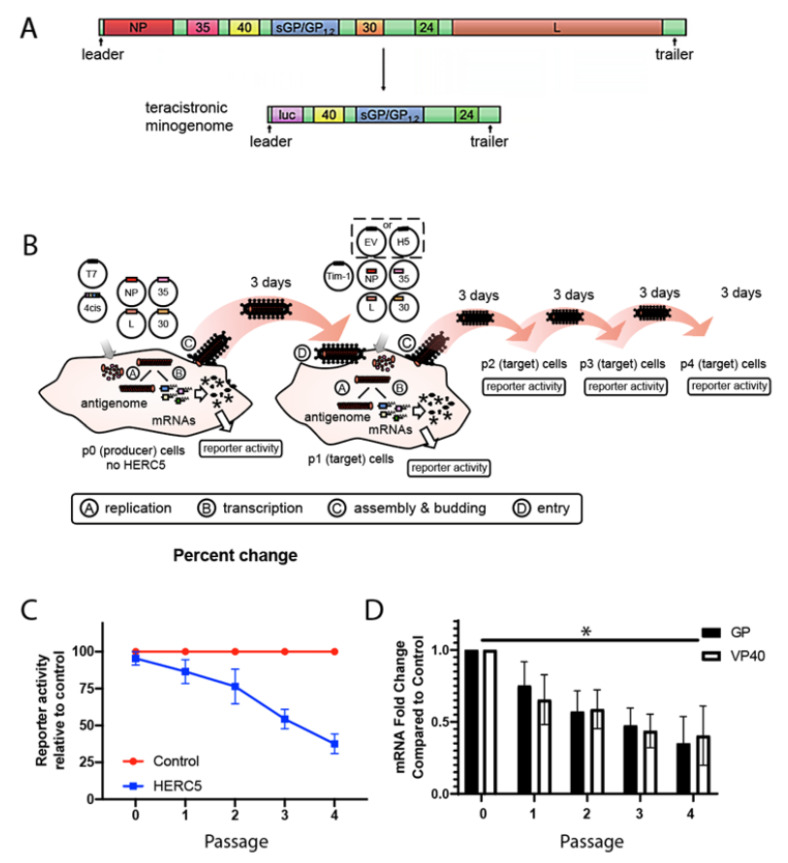
HERC5 inhibits EBOV trVLP replication. (**A**) Schematic depicting EBOV full-length genome and the derived tetracistronic minigenome. (**B**) The trVLP propagation assay. A tetracistronic EBOV minigenome (4cis) is expressed in cells together with the viral ribonucleoprotein complex (RNP) proteins (NP, VP35, VP30 and L). After the initial transcription by a co-expressed T7 polymerase, the minigenome is replicated and transcribed by the RNP proteins. Expression of VP40, GP and VP24 from the minigenome leads to the formation of infectious trVLPs containing minigenomes, which can infect target cells. Multiple infectious cycles can be modeled in cells expressing NP, VP35, L, VP30 and Tim-1 without the need for additional transfections of plasmids carrying VP40, GP and VP24. The figure was adapted from (Watt et al., 2014), copyright © American Society for Microbiology, J. Virol. 88, 2014, 10,511–10,524, doi:10.1128/JVI.01272-14. (**C**) Quantification of trVLP propagation in the presence and absence of HERC5. The trVLP propagation assay was performed using tetracistronic minigenomes carrying a luciferase reporter, EBOV VP40, VP24 and EBOV GP over four passages (spanning 12 days). All EBOV minigenomes and plasmids carrying the EBOV proteins are based on EBOV *H. sapiens*-tc/COD/1976/Yambuku-Mayinga. Luciferase reporter activity relative to the control (trVLPs propagated in the absence of HERC5) is shown. The data shown represent the average (+/− S.E.M.) of four independent experiments. Linear regression analysis, F = 39.14. DFn = 1, DFd = 36; *p* < 0.0001. (**D**) The mRNA of GP and VP40 was measured using qRT-PCR at each passage. The data shown represent the average (+/− S.E.M.) of the four independent experiments represented in part C. * *p* < 0.05; One-way ANOVA with Dunnet’s multiple comparisons test compared to the control.

Previous studies have identified HERC5 as a potent inhibitor of diverse viruses [30,31,32,33,34,35,36]. To determine if HERC5 restricts EBOV particle production and replication, we used an EBOV (Zaire) transcription- and replication-competent VLP (trVLP) system. This system utilizes a tetracistronic minigenome (‘4cis’) carrying a *luciferase* reporter gene together with *VP40*, *VP24*, and *GP* (Figure 1A) [40,45]. The advantage of this system over conventional VLP assays is that the viral proteins VP40, GP and VP24 are encoded by the minigenome and expressed from the EBOV promoter in a more natively regulated fashion [40]. The co-expression of this minigenome with NP, VP35, VP30, and L drive genome replication and transcription, synthesis of the minigenome-encoded proteins, and formation of infectious trVLPs. These trVLPs incorporate minigenomes and are capable of undergoing multiple rounds of replication and infection in target cells that express NP, VP35, VP30, L and Tim-1 (Figure 1B). The replication of these trVLPs was quantified over multiple passages (every three days) by measuring the luciferase reporter activity within cells. As a negative control, the plasmid carrying the Ebola *L* gene was omitted from the transfections, which abrogated the trVLP formation. Compared to the control cells transfected with an empty vector plasmid, cells expressing HERC5 exhibited a significant reduction in trVLP replication over four passages (Figure 1C). The reduction in luciferase reporter activity also correlated with a reduction in GP and VP40 mRNA levels (Figure 1D). 

### 3.2. HERC5 Inhibits EBOV VP40 Particle Production

**Figure 2:**
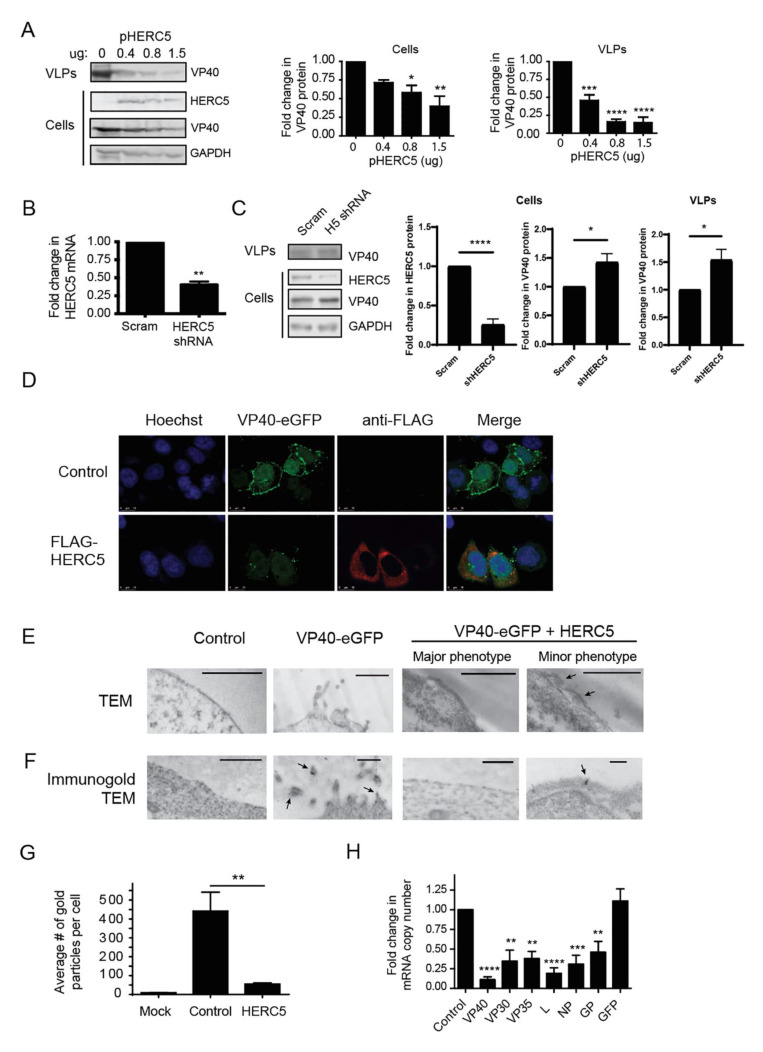
HERC5 inhibits EBOV VP40 particle production. (**A**) 293T cells were co-transfected with plasmids carrying FLAG-tagged VP40 (pFLAG-VP40) and increasing concentrations of FLAG-tagged HERC5 (pFLAG-HERC5). Empty vector plasmid was transfected in the condition with no HERC5 and used to ensure equal amounts of DNA were transfected in each condition. Forty-eight hours post-transfection, purified VLPs released into the cell supernatant and intracellular protein were subjected to quantitative Western blot analysis using anti-FLAG, anti-VP40 and anti-GAPDH. The average densitometric quantification of VP40 protein bands is shown to the right after normalization to GAPDH levels (+/− S.E.M.). A representative Western blot of four independent experiments is shown. (**B**) 293T cells were co-transfected with pFLAG-VP40 and either scrambled short-hairpin RNA (shRNA) (scram) or HERC5_shRNA_ (shHERC5). Forty-eight hours after transfection, intracellular levels of HERC5 mRNA were quantified via qPCR. Data shown is the average (+/− S.E.M.) of three independent experiments. (**C**) 293T cells were transfected with either scrambled short-hairpin RNA (shRNA) (scram) or HERC5_shRNA_ (shHERC5) for 24 h and then with pFLAG-HERC5 and pFLAG-VP40 for forty-eight hours. Purified VLPs released into the cell supernatant and intracellular protein were subjected to quantitative Western blot analysis using anti-FLAG and anti-GAPDH. The average densitometric quantification of VP40 protein bands is shown to the right after normalization to GAPDH levels (+/− S.E.M.). A representative Western blot of four independent experiments is shown. (**D**) HeLa cells were co-transfected with pVP40-EGFP and either empty vector (control) or pFLAG-HERC5 and visualized using confocal microscopy 48 h post-transfection. (**E**) 293T cells were “mock” transfected (control), transfected with empty vector and pVP40-EGFP, or transfected with pFLAG-HERC5 and pVP40-EGFP and analyzed via transmission electron microscopy (TEM) after 48 h. Virus particles beneath the plasma membrane are indicated with arrows. (**F**) Representative immunogold TEM images of 293T cells transfected as in (**E**) and labelled with 5 (+/− 2) nm anti-GFP immunogold particles. Immunogold-labelled VLPs are indicated with arrows. Scale bars = 500 nm. (**G**) The number of gold particles per positive cell was counted and presented as the average number of particles per cell (+/− S.E.M). (**H**) 293T cells were co-transfected with plasmids carrying FLAG-HERC5 (or empty vector) and either EBOV VP40, VP30, VP35, L, NP, GP or GFP at a ratio of 10:1 (HERC5: EBOV plasmids). Forty-eight hours post-transfection viral mRNA was measured using qPCR after normalization to GAPDH mRNA levels. Data shown are representative of three independent experiments (+/− S.E.M.). **** *p* < 0.0001, *** *p* < 0.001, ** *p* < 0.01, * *p* < 0.05; One-way ANOVA with Dunnet’s multiple comparisons test compared to the control (**A**,**G**); Student’s paired t-test (**B**,**C**,**H**).

Previous studies showed that HERC5 interferes with the function of key viral structural proteins [30,31,35,36]. The EBOV structural protein VP40 is necessary and sufficient for the assembly and budding of virus particles. When expressed in the absence of any other viral protein, VP40 can form VLPs that bud and are released from cells similar to wild-type EBOV [46,47,48]. To determine if HERC5 targets VP40, we co-transfected 293T cells with a plasmid carrying VP40 and increasing concentrations of plasmids carrying either empty vector control or FLAG-tagged HERC5. VP40 protein levels within cells and in VLPs were measured using quantitative Western blotting. HERC5 transfection did not alter cell viability (Figure S1A). As shown in Figure 2A and Figure S1B,C, HERC5 inhibited the production of VP40 VLPs in a dose-dependent manner when VP40 is tagged with either GFP or with FLAG but had no effect on intracellular GFP levels. As a control, transfection with *HERC4*, a closely related member of the small *HERC* family, did not significantly alter cell viability, VP40 or GFP levels (Figure S1D–F). In contrast, when *HERC5* mRNA levels were reduced using RNA interference, an increase in intracellular VP40 protein levels and an increase in the production of VP40 VLPs were observed compared to the control cells (Figure 2B,C).

We also assessed the impact of HERC5 expression on VLPs using confocal microscopy and transmission electron microscopy (TEM). As expected, cells expressing VP40 with enhanced green fluorescent protein fused at its amino-terminus (VP40-EGFP) exhibited punctate fluorescence at the cell surface (Figure 2D). In contrast, cells co-expressing VP40-EGFP and HERC5 exhibited substantially less punctate fluorescence at the cell surface compared to the control cells. The presence of VP40 protein at the cell surface was also confirmed using TEM and immunogold TEM (Figure 2E,F). In cells expressing VP40-EGFP alone, an accumulation of immunogold particles was observed in budding structures at the cell surface, which was significantly different from a random distribution (Tables S1 and S2). Cells expressing HERC5 exhibited markedly fewer VP40-EGFP-containing structures at the cell surface compared to the control cells. In addition, cells expressing HERC5 exhibited on average eight-fold fewer immunogold particles per cell compared to the control cells (Figure 2G). Notably, the few VP40-EGFP-containing structures that were observed in cells expressing HERC5 were located predominantly in a region under the plasma membrane. 

We then asked whether the reduced VP40 protein levels correlated with reduced intracellular VP40 mRNA levels. The quantitative PCR showed that 293T cells co-expressing HERC5 and FLAG-tagged VP40 exhibited reduced intracellular levels of VP40 mRNA (nine-fold) compared to the control cells not expressing HERC5 (Figure 2H). Similar results were obtained when HERC5 was co-expressed with a VP40-EGFP fusion protein (Figure S2). As a control, HERC5 expression had no significant effect on EGFP mRNA levels when EGFP was expressed alone (Figure 2H and Figure S2). To determine if the effect of HERC5 is specific for VP40 mRNA, we assessed the impact of HERC5 expression on the level of other EBOV mRNAs. Cells co-expressing HERC5 and either VP30, VP35, L or NP exhibited a two- to five-fold reduction in mRNA levels compared to the control cells (Figure 2H). Together, these data show that HERC5 inhibits EBOV VP40 particle production by a mechanism involving the depletion of EBOV mRNAs.

### 3.3. HERC5 RLD Is Necessary and Sufficient for Inhibition of VP40 Particle Production

**Figure 3:**
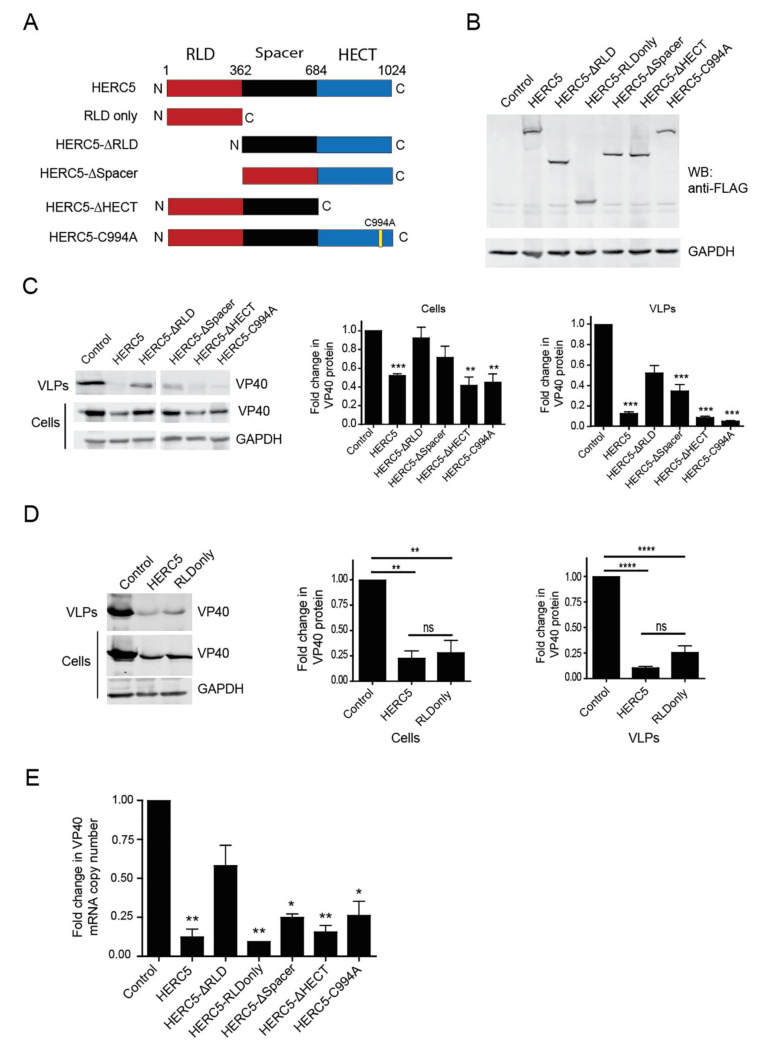
The RLD is necessary and sufficient for HERC5-mediated restriction. (**A**) Schematic of the different HERC5 mutant constructs. (**B**) Representative Western blot showing consistent expression of wild-type HERC5 and mutant forms of HERC5. 293T cells were transfected with either empty vector or plasmids carrying FLAG-tagged HERC5, HERC5-ΔRLD, HERC5-RLDonly, HERC5-ΔSpacer, HERC5-ΔHECT or HERC5-C994A. Forty-eight hours after transfection, cell lysate was subjected to Western blot analysis using anti-FLAG and anti-GAPDH. (**C**) 293T cells were co-transfected with plasmids carrying FLAG-tagged VP40 and either empty vector, wild-type HERC5 or one of the HERC5 mutants listed in (**A**). Forty-eight hours post-transfection, purified VLPs released into the supernatant and intracellular protein were examined by Western blotting using anti-FLAG and anti-GAPDH. VP40 protein levels were quantified densitometrically after normalization to GAPDH levels (graphs on the right). (**D**) 293T cells were co-transfected with plasmids carrying VP40-EGFP and either empty vector, HERC5 or HERC5-RLDonly. Cell lysates and VLPs were analyzed via Western blotting using anti-GFP and anti-GAPDH. VP40-EGFP protein levels were quantified densitometrically (graphs on the right). (**E**) 293T cells were co-transfected with plasmids carrying FLAG-tagged VP40 and either empty vector, HERC5, HERC5-ΔRLD, HERC5-RLDonly, HERC5-ΔSpacer, HERC5-ΔHECT or HERC5-C994A. Forty-eight hours post-transfection, mRNA was isolated and used to measure intracellular VP40 mRNA levels using qPCR. All data shown are representative of three independent experiments (+/− S.E.M.). **** *p* < 0.0001, *** *p* < 0.001, ** *p* < 0.01, * *p* ≤ 0.05, ns (not significant) *p* > 0.05; One-way ANOVA with Dunnet’s multiple comparisons test compared to the control.

To determine if the RLD or HECT domains of HERC5 are required for inhibition, we tested the ability of several HERC5 mutants to inhibit VP40 particle production. 293T cells were co-transfected with plasmids carrying VP40 and either empty vector (control), wild type HERC5 or HERC5 mutants lacking the RCC1-like domain (HERC5-ΔRLD), spacer region (HERC5-Δspacer) or HECT domain (HERC5-ΔHECT). We also tested the HERC5 RLD alone (HERC5-RLDonly) or HERC5 containing a cysteine to an alanine point mutation of residue 994 (HERC5-C994A), which specifically inactivates its E3 ligase activity (Figure 3A). Each of the FLAG-tagged mutant proteins was expressed at similar levels in 293T cells (Figure 3B).

As shown in Figure 3C, cells expressing wild type HERC5, HERC5-ΔHECT or HERC5-C994A reduced VP40 protein levels, which also correlated with reduced VP40 VLP production. In contrast, cells expressing HERC5-ΔRLD, and to a lesser extent HERC5-Δspacer, exhibited a diminished capacity to reduce VP40 protein levels and VP40 VLP production. Notably, expression of the HERC5 RLD alone (HERC5-RLDonly) reduced VP40 protein levels and VP40 VLP production similar to wild-type HERC5 (Figure 3D). We also examined the ability of the different HERC5 mutants to reduce VP40 mRNA levels. All HERC5 mutants except for HERC5-ΔRLD significantly reduced VP40 mRNA levels (Figure 3E). Taken together, these data show that the HERC5 RLD is necessary and sufficient to reduce VP40 mRNA levels and VP40 particle production.

### 3.4. HERC5 Depletes VP40 mRNA Independently of ZAP

**Figure 4:**
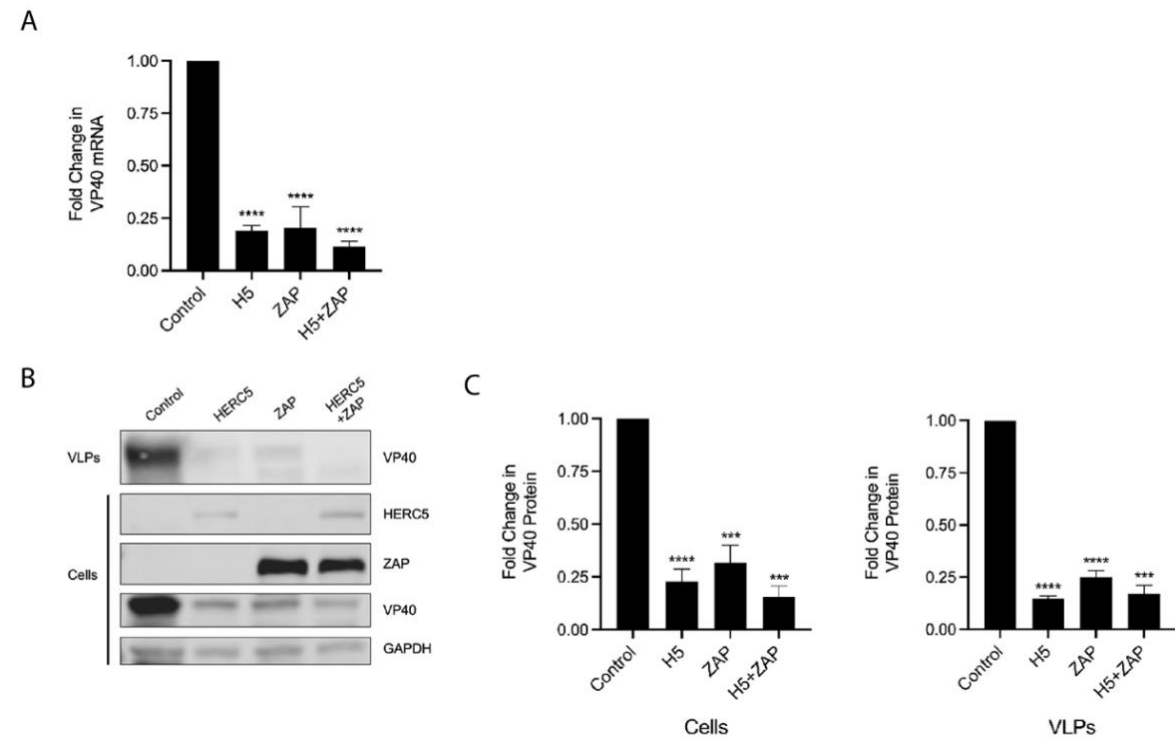
HERC5 restricts VP40 independently of ZAP. 293T ZAP knockout cells were co-transfected with plasmids carrying FLAG-tagged VP40 and either empty vector control, HERC5, ZAP (short isoform), or HERC5 and ZAP (short isoform). Twenty-four hours post-transfection, cell lysates and VLP-containing supernatants were harvested. (**A**) Intracellular VP40 mRNA levels were measured using qPCR and normalized to GAPDH. The data shown are representative of four independent experiments. (**B**) Purified VLPs released into the cell supernatant and intracellular proteins were subjected to Western blot analysis using anti-FLAG and anti-GAPDH. Representative Western blot of three independent experiments is shown. (**C**) The average densitometric quantification of VP40 protein bands from B is shown after normalization to GAPDH levels. Results are presented as mean (± SEM) fold changes in VP40 protein or mRNA. **** *p* < 0.001, *** *p* < 0.001, One-way ANOVA with Tukey’s multiple comparisons test.

ZAP (also called Zinc finger CCCH-type, antiviral 1, ZC3HAV1, and Poly (ADP-ribose) polymerase 13, PARP13) is an antiviral protein that causes significant loss of viral mRNAs from evolutionarily diverse RNA viruses, including *Filoviridae, Retroviridae, Togaviridae* and *Hepadnaviridae* [49,50,51,52,53,54,55]. We, therefore, asked if ZAP was required for HERC5-mediated depletion of EBOV mRNA. We co-expressed VP40 and HERC5 in 293T cells that were knocked out for all ZAP isoforms and measured VP40 mRNA and protein levels using qPCR and Western blotting [56,57]. Cells expressing HERC5 in the absence of ZAP significantly reduced VP40 mRNA levels (Figure 4A). Exogenous expression of ZAP (short isoform) in the ZAP knockout cells reduced VP40 mRNA levels as previously shown [52,56]. Co-expression of HERC5 and ZAP together resulted in an enhanced loss of VP40 mRNA (Figure 4A). In support of this observation, cells expressing HERC5 in the absence of ZAP significantly reduced intracellular VP40 protein and VP40 VLPs the cell supernatant (Figure 4B,C). Together, these data show that ZAP is not required for HERC5-mediated reduction of VP40 mRNA.

### 3.5. EBOV GP and L Proteins Antagonize HERC5

**Figure 5:**
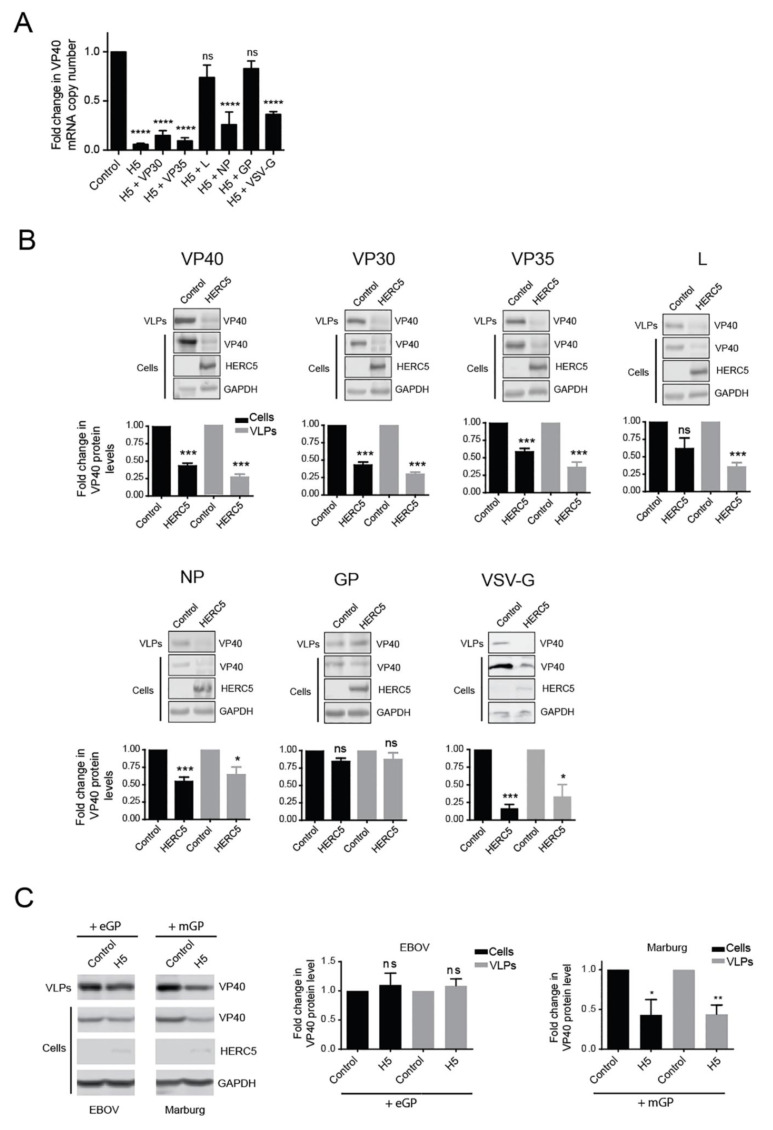
EBOV GP and L antagonize HERC5. 293T cells were co-transfected with plasmids carrying FLAG-tagged VP40 and either empty vector or HERC5 and one plasmid carrying either EBOV VP30, VP35, L NP, GP or VSV-G. Forty-eight hours post-transfection, VP40 mRNA was measured using qPCR (**A**) and VP40 protein levels in cell lysates and VLPs released into supernatant were analyzed by quantitative Western blotting and quantified densitometrically after normalization to GAPDH levels (**B**). (**C**) 293T cells were co-transfected with plasmids carrying FLAG-tagged VP40 and either empty vector or HERC5, and one of EBOV GP (eGP) or MARV GP (mGP). Forty-eight hours post-transfection, VP40 protein levels in cell lysates and VLPs released into the supernatant were analyzed via Western blotting using anti-FLAG and anti-GAPDH. The data shown represent the average (+/− S.E.M.) of three independent experiments. * *p* < 0.05, ** *p* < 0.01, *** *p* < 0.001, **** *p* < 0.0001, ns (not significant) *p* > 0.05; One-way ANOVA with Dunnett’s multiple comparisons test compared to the control (**A**); Student’s paired t-test (**B**,**C**).

Despite an early and robust IFN-signaling response to EBOV infection, EBOV proteins ultimately suppress this response leading to pathogenesis [2,3,4,5,6,7,8,9,10,11,12]. Given the potent antiviral activity of HERC5 towards EBOV mRNAs, we asked if any of the EBOV proteins could antagonize this activity. VP40 mRNA levels in cells co-expressing HERC5 and various EBOV proteins were measured by qPCR. As shown in Figure 5A, VP40 mRNA levels were rescued in cells co-expressing GP or L protein, but not VP30, VP35, NP or the non-EBOV protein vesicular stomatitis virus-G (VSV-G) protein. Western blot analysis of cell lysates correlated with the qPCR data where only L and GP proteins rescued intracellular VP40 protein levels (Figure 5B). Western blot analysis of VP40 VLPs in the supernatant revealed that GP but not L protein rescued VLP production, indicating that only GP was able to fully rescue VLP production. 

To determine if the ability of EBOV GP to antagonize HERC5 is specific to the *Ebolavirus* genus, we tested the ability of Marburg virus (MARV) GP, which belongs to the *Marburgvirus* genus, to antagonize HERC5. In contrast with EBOV GP, co-expression of MARV GP failed to rescue VP40 VLP production (Figure 5C). Together these data show that EBOV GP antagonizes HERC5 activity and that this antagonism does not appear to be conserved between filovirus genera.

### 3.6. EBOV and MARV GP Differentially Antagonize HERC5 Inhibition of EBOV trVLP Replication

**Figure 6:**
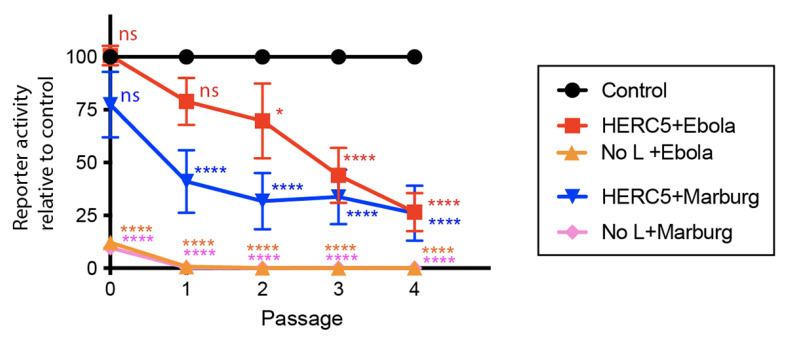
EBOV GP and MARV GP differentially antagonize HERC5. Quantification of trVLP propagation in the presence and absence of HERC5. The trVLP propagation assay was performed using tetracistronic minigenomes carrying a luciferase reporter, EBOV VP40, VP24 and either EBOV GP or MARV GP over four passages (spanning 12 days). All EBOV minigenomes and plasmids carrying the EBOV proteins are based on EBOV *H. sapiens*-tc/COD/1976/Yambuku-Mayinga. As a negative control (‘No L’), the plasmid carrying the Ebola *L* gene was omitted from the transfections. Luciferase reporter activity relative to the control (trVLPs propagated in the absence of HERC5) is shown. The data shown represent the average (+/− S.E.M.) of at least six independent experiments. **** *p* < 0.0001, * *p* ≤ 0.05, ns (not significant) *p* > 0.05; Two-way ANOVA with Dunnett’s multiple comparisons test compared to the no HERC5 control.

We utilized the EBOV trVLP system described in Figure 1 to determine if genus-specific GP (EBOV or MARV) could antagonize the ability of HERC5 to inhibit trVLP replication. To test the effect of different GPs on trVLP replication, two different sets of trVLP particles were generated at P0. One set contained EBOV GP (trVLP_EBOV GP_) and was generated as described in Figure 1A. The second set was generated in an identical way except that the EBOV *GP* gene in the ‘4cis’ plasmid minigenome was substituted with the MARV *GP* gene (trVLP_MARV GP_). This allowed us to test the impact of different GPs in the VLPs while maintaining the same background of EBOV proteins. As a negative control, the plasmid carrying the Ebola *L* gene was omitted from the transfections, which abrogates trVLP formation. Compared to the control cells not expressing HERC5, cells expressing HERC5 exhibited significantly reduced levels of trVLP_MARV GP_ and trVLP_EBOV GP_ replication over four passages (spanning 12 days) (Figure 6). Notably, HERC5 inhibited trVLP_MARV GP_ replication significantly more than trVLP_EBOV GP_ replication over two passages (*p* < 0.01, Two-way ANOVA). Together, these data show that EBOV GP and MARV GP differentially antagonize HERC5 inhibition of EBOV trVLP replication. 

## 4. Discussion

Hundreds of IFN-induced proteins are part of the early and robust immune response to EBOV infection in primates [2,3,4,5,6,7,8,9,10,11,12]. Characterization of the key effector proteins of this defense and how EBOV overcomes them will provide a better understanding of the virus–host interactions that occur early in infection. HERC5 is one of the most up-regulated antiviral proteins in the early response to EBOV infection in vivo; however, its role in EBOV replication was previously unknown [3,5,6,29]. 

In this study, we showed that HERC5 inhibits EBOV VLP replication via a novel E3 ligase-independent mechanism. This mechanism involves the depletion of viral mRNAs and requires the RLD domain of HERC5. We previously showed that HERC5 inhibits the nuclear export of HIV-1 RNA genomes by a different E3 ligase-independent mechanism, one that also requires the RLD domain of HERC5 [30]. These E3 ligase-independent antiviral activities, together with its well-documented E3 ligase-dependent antiviral activities [58], identifies HERC5 as a multifunctional antiviral protein. It is perhaps not surprising that HERC5 has evolved multiple mechanisms of restriction of viruses. The ancestral *HERC* gene is believed to have arisen from a gene fusion event between an *RCC1*-like gene and a *HECT* gene [59,60]. This fusion event gave rise to a family of small HERC proteins containing an amino-terminal RLD and a carboxyl-terminal HECT domain that is highly conserved among vertebrates spanning >595 million years of evolution [36,59,60]. Moreover, HERC5 has been evolving under strong positive selection, which is characteristic of many host restriction factors involved in an evolutionary struggle with viruses [30,60,61]. The ability of HERC5 to inhibit viruses via both E3 ligase-dependent and -independent mechanisms would confer a strong evolutionary advantage to its host, making it more difficult for viruses to evolve countermeasures to HERC5. 

Like HERC5, ZAP is present in evolutionarily diverse vertebrates and has evolved under strong positive selection [30,36,62]. ZAP targets diverse viruses such as HIV-1, MoLV and XMRV (*Retroviridae*), Ebola and Marburg viruses (*Filoviridae*), alphavirus, Sindbis, Semliki Forest and Ross River viruses (*Togaviridae*), hepatitis B virus (*Hepadnaviridae*) and double-stranded DNA murine gamma herpesvirus (*Herpesviridae*) [49,50,51,52,53,54,55,63]. ZAP is known to inhibit a wide range of antiviral activities, including recruiting the exosome complex to target viral RNAs for degradation [49,51,53,55,57,64,65,66,67,68]. ZAP also exhibits virus specificity since it has no antiviral effect on vesicular stomatitis, poliovirus, yellow fever and herpes simplex I viruses [49]. We showed here that HERC5 depletes EBOV mRNAs in a ZAP-independent manner. Our finding that the HERC5 RLD is necessary and sufficient for EBOV mRNA depletion further supports an E3 ligase-independent mechanism of restriction. It was previously shown that the RLD is required for the association of HERC5 with polyribosomes [35]. It is possible that HERC5 exploits this interaction to recruit other RNA degradation machinery to EBOV mRNAs. 

Although we showed that the RLD alone was necessary and sufficient to inhibit particle production, HERC5 lacking the RLD failed to completely inhibit VP40 VLP particle production. Since the RLD is important but not essential for its E3 ligase activity, it is possible that the E3 ligase activity of HERC5 also confers some antiviral activity towards VLP production via ISGylation of viral and/or host proteins involved in particle production [31,35,38]. It was previously shown that over-expression of ISG15 alone inhibited budding of EBOV VP40 VLPs by disrupting Nedd4 function and subsequent ubiquitination of VP40, which is necessary for viral egress [69]. It is unknown whether HERC5 was involved in this activity since it was not investigated. Although our data show that the predominant mechanism by which HERC5 inhibits EBOV VLP production involves the depletion of EBOV mRNAs, visual inspection of cells co-expressing EBOV VP40 and HERC5 by TEM and confocal microscopy revealed an accumulation of the VP40 protein at the localized regions in the plasma membrane in some cells, consistent with the idea of a second mechanism of inhibition acting later in particle production. HERC5-induced trapping of virus particles at the plasma membrane has also been observed with HIV-1 [31]. However, it is also possible that these accumulations represent particles in the process of budding that have escaped HERC5 restriction. HERC5 reduced intracellular mRNA levels of viral protein expressed both from a plasmid system (Figure 2H) and of viral mRNA expressed from a tetracistronic minigenome. It is unknown how HERC5 can target viral RNAs but not non-viral RNA such as GFP. Perhaps virus-specific RNA sequences recruit HERC5 and/or RNA depletion machinery similar to how ZAP selectively recognizes high CpG-containing viral RNA. Further studies are needed to decipher this novel antiviral function of HERC5.

Animal model studies have suggested that the Type I IFN response plays an important role in restricting EBOV replication and that the ability of EBOV to overcome this response may be a requirement for lethal infection [70,71]. Although EBOV VP24 and VP35 can act broadly to dampen the IFN response, several IFN-induced antiviral proteins, including HERC5, are also highly upregulated early in response to other stimuli associated with infection, such as pro-inflammatory cytokines [72,73,74]. As such, it is likely that EBOV evolved additional antagonists of such antiviral proteins. Indeed, EBOV GP can directly antagonize the restriction factor BST-2/tetherin without altering BST-2/tetherin expression levels or cellular localization [24,75,76,77,78,79,80,81,82]. As shown herein, EBOV GP also antagonizes HERC5 without altering HERC5 expression levels. Although controversial, GP sequence diversity has been shown to affect EBOV transmission and virulence, as demonstrated in the 2013-2016 EBOV epidemic [83,84]. We showed here that variations in GP sequence, such as those found between different filovirus genera (e.g., EBOV and MARV), also influence the potency of antagonism of HERC5 during the early stages of EBOV trVLP replication. It is unclear how GP, which is predominantly localized to the plasma membrane, can rescue EBOV mRNA levels. GP expression is known to alter the expression and trafficking of select cellular proteins; therefore, it is possible that proteins involved in viral RNA stability are affected by GP expression [85,86,87]. Important next steps will be to characterize the mechanism of GP antagonism and to test the importance of this HERC5-GP axis early in infection using animal models.

It is interesting that EBOV L protein was also able to rescue HERC5-induced VP40 mRNA depletion but unable to antagonize the release of VP40 VLPs into the cell supernatant. The mechanism underlying this antagonism is not fully understood; however, it was previously shown that L protein antagonizes ZAP [52]. It is possible that L protein also specifically antagonizes HERC5-induced depletion of mRNAs. However, we speculate that the E3 ligase activity of HERC5 remains functional, leading to the ISGylation of viral and/or host proteins and subsequent arrest of later steps in viral particle production.

In conclusion, we showed that HERC5 inhibits EBOV virus particle production by a mechanism involving the depletion of EBOV mRNAs. Our data also identifies a novel ‘protagonist–antagonistic’ relationship between HERC5 and GP early in EBOV infection. With the ability to inhibit HERC5 and other restriction factors, GP is an attractive target for the development of small molecule compounds that interfere with this antagonism.

## Data Availability

Not applicable.

## Supplementary Materials

The following are available online at https://www.mdpi.com/article/10.3390/cells10092399/s1, Figure S1: HERC5 depletes GFP- and FLAG-tagged VP40 mRNA but not GFP mRNA, Figure S2: HERC5 depletes GFP- and FLAG-tagged VP40 mRNA but not GFP mRNA, Table S1: Quantification of 5nm gold particle-labeled anti-GFP in cells expressing empty vector and VP40-EGFP, Table S2: Quantification of 5nm gold particle-labeled anti-GFP in cells expressing HERC5 and VP40-EGFP.
