# Supplementary material for: Interferon-Induced HERC5 Inhibits Ebola Virus Particle Production and Is Antagonized by Ebola Glycoprotein
Source: Cells. 2021 Sep 13;10(9):2399. doi: 10.3390/cells10092399 (PMC8472148; doi:10.3390/cells10092399)
Supplement: Supplementary file 1 [file cells-10-02399-s001.zip › cells-1221569-supplementary.pdf]

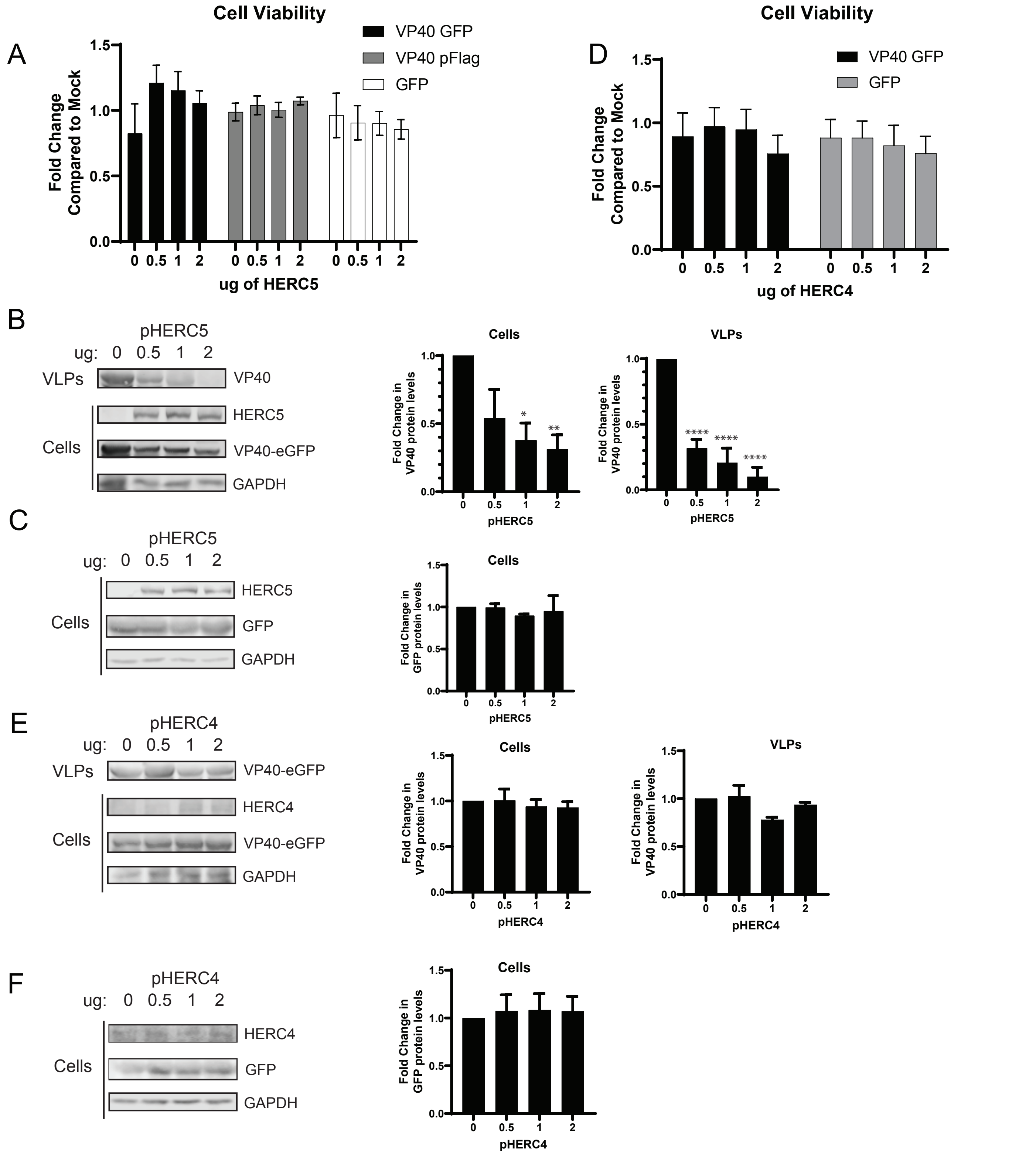

Figure S1: HERC5 depletes GFP- and FLAG-tagged VP40 mRNA but not GFP mRNA. 293T cells were co-transfected with plasmids carrying FLAG-tagged VP40 (pFLAG-VP40), VP40-eGFP or just GFP plasmid and increasing concentrations of FLAG-tagged HERC5 (pFLAG-HERC5). Empty vector plasmid was transfected in the condition with 0 ug of HERC5 plasmid and used to ensure equal amounts of DNA were transfected in each condition Forty-eight hours post-transfection cell viability was measured using the CCK8 assay. Fold change in absorbance compared to untransfected cells was calculated. Results of 4 independent experiments are shown (B) 293T cells were co-transfected with plasmids carrying VP40-eGFP or just GFP plasmid and increasing concentrations of FLAG-tagged HERC4 (pFLAG-HERC4). Forty-eight hours post-transfection cell viability was measured using CCK8 assay. Fold change in absorbance compared to untransfected cells was calculated. Results of 4 independent experiments are shown. (C and D) Cells were transfected as in (A) purified VLPs released into the cell supernatant and intracellular protein were subjected to Western blot analysis using anti-FLAG, anti-VP40 and anti-GAPDH. The average densitometric quantification of VP40 protein bands is shown to the right after normalization to GAPDH lev-els (+/- S.E.M.). Representative Western blot of four independent experiments is shown. (E and F) Cells were transfected as in (B) purified VLPs released into the cell supernatant and intracellular protein were subjected to Western blot analysis using anti-HERC4, anti-VP40 and anti-GAPDH. The average densitometric quantification of VP40 protein bands is shown to the right after normalization to GAPDH levels (+/- S.E.M.). Representative Western blot of four independent experiments is shown.

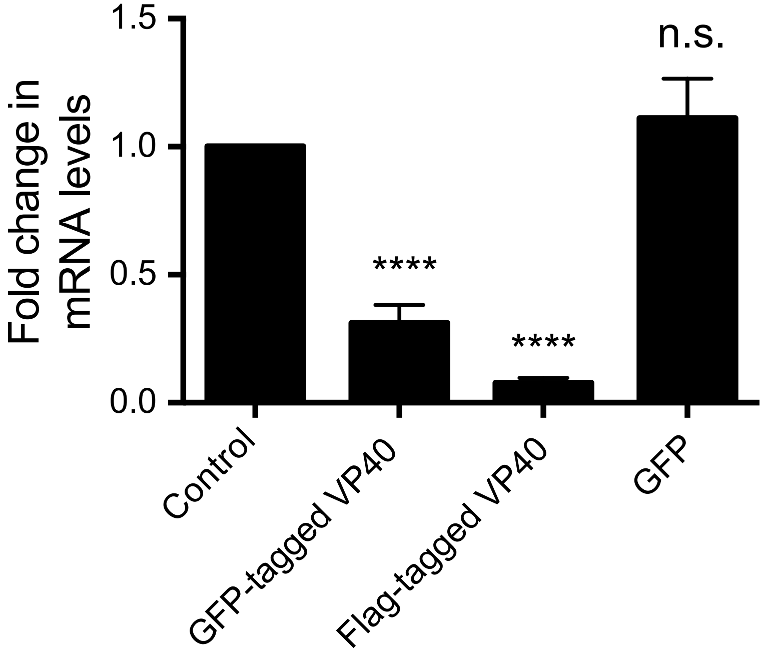

Figure S2: HERC5 depletes GFP- and FLAG-tagged VP40 mRNA but not GFP mRNA

Table S1: Quantification of 5nm gold particle-labeled anti-GFP in cells expressing empty vector and VP40-eGFP.

**Region**	**Observed gold count, G_0**	**Point count, P**	**Expected gold count, G_e**	**G_0/G_e**	X^2	**X^2 as %**
**Particles + plasma membrane**	112	17	14	7.73	656.78	79.28
**Cytoplasm + nucleus**	99	64	55	1.82	36.33	4.39
**Non-particle + non-cell**	7	175	149	0.05	135.35	16.34
**TOTAL**	218	256	218		828.46	100

For *X2*= 656.78 and df=2, P <0.0001 (*X2* analysis). The gold labeling distribution is significantly different from random. Only the particles+plasma membrane region (Go/Ge= 7.73, *X2*= 79.3% of total) meets the two criteria for being preferentially labeled ((Go/Ge) ≥ 1 and *X2* > 10% of total).

Table S2: Quantification of 5nm gold particle-labeled anti-GFP in cells expressing HERC5 and VP40-eGFP.

Region	Observed gold count, G_0	Point count, P	Expected gold count, G_e	G_0/G_e	X^2	X^2 as %
**Particles + plasma membrane**	30	15	4	7.75	176.43	66.94
**Cytoplasm + nucleus**	35	49	13	0.71	39.62	15.03
**Non-particle + non-cell**	1	192	53	0.02	47.52	18.02
**TOTAL**	70	256	70		263.57	100

For *X2*= 176.43 and df=2, P <0.0001 (*X2* analysis). The gold labeling distribution is significantly different from random. Only the particles+plasma membrane region (Go/Ge= 7.75, *X2*= 66.9% of total) meets the two criteria for being preferentially labeled ((Go/Ge) ≥ 1 and *X2* > 10% of total).
